# Improving Fracture Toughness of Tetrafunctional Epoxy with Functionalized 2D Molybdenum Disulfide Nanosheets

**DOI:** 10.3390/polym13244440

**Published:** 2021-12-17

**Authors:** Megha Sahu, Lakshmi Narasimhan, Ashok M. Raichur, Alexandru Sover, Romeo C. Ciobanu, Nicolae Lucanu, Mihaela Aradoaei

**Affiliations:** 1 Department of Materials Engineering, Indian Institute of Science, Bengaluru 560012, India; megha.4sahu@gmail.com (M.S.); narasimhanbiochem@gmail.com (L.N.); 2 Department of Technology, Technical Faculty, Ansbach University of Applied Sciences, 91522 Ansbach, Germany; a.sover@hs-ansbach.de; 3 Department of Electrical Measurements and Materials, Faculty of Electrical Engineering, Technical University Gh. Asachi Iasi, 700050 Iasi, Romania; rciobanu@yahoo.com (R.C.C.); nicolae.lucanu@academic.tuiasi.ro (N.L.); mihaela.aradoaei@academic.tuiasi.ro (M.A.)

**Keywords:** polymer-matrix composites (PMCs), mechanical testing, thermosetting resin, exfoliated molybdenum disulfide, fracture toughness, epoxy toughening, 2D transition metal dichalcogenides, layered nanomaterials

## Abstract

In this work, improved fracture toughness of tetra-functional epoxy polymer was obtained using two-dimensional (2H polytype) molybdenum disulfide (MoS_2_) nano-platelets as a filler. Simultaneous in-situ exfoliation and functionalization of MoS_2_ were achieved in the presence of cetyltrimethylammonium bromide (CTAB) via sonication. The aim was to improve the dispersion of MoS_2_ nanoplatelets in epoxy and enhance the interfacial interaction between nanoplatelets and epoxy matrix. Epoxy nanocomposites with CTAB functionalized MoS_2_ (f-MoS_2_) nanoplatelets, ranging in content from 0.1 wt% up to 1 wt%, were fabricated. Modified MoS_2_ improved the fracture properties (81%) of tetrafunctional epoxy nanocomposites. The flexural strength and compressive strength improved by 64% and 47%, respectively, with 0.25 wt% loading of f-MoS_2_ nanoplatelets compared to neat epoxy. The addition of f-MoS_2_ nanoplatelets enhanced the thermomechanical properties of epoxy. This work demonstrated the potential of organically modified MoS_2_ nanoplatelets for improving the fracture and thermal behavior of tetrafunctional epoxy nanocomposites.

## 1. Introduction

Tetrafunctional epoxy resins are among the most critical high-performance thermosetting polymers. These epoxy resins offer excellent strength and heat resistance for advanced structural and high-temperature applications in aerospace industries. The last decade has seen the increasing use of epoxy-based composites in the aerospace industry, especially in the Boeing 787 and Airbus A350. In these aircrafts, the fuselage, wings, and empennage, which are major structural parts, are manufactured using epoxy-based fiber reinforced composites. Due to their excellent resistance to fatigue and corrosion as well as enabling of a reduction in the number of components, more and more structural parts in aircraft are being designed using epoxy-based composites. However, the high crosslink density of epoxy polymers is low, which leads to decreased resistance to crack propagation. To address this problem, toughening agents that enhance the fracture properties of tetrafunctional epoxy without compromising modulus and thermal properties are required. There is little work reported on improving the fracture properties of tetrafunctional epoxy polymer. Most of the published work is on bifunctional epoxy polymers for low-temperature applications. Graphene and its derivatives provide excellent reinforcement for epoxy polymers and enhance their thermal properties at low concentrations [1,2]. However, because of the highly conducting nature of graphene [3], its usage as a filler is restricted when polymer composites with unaltered insulating properties are required [4], e.g., the structures which come in contact with the metal foil used for lightning strike protection. The presence of a conducting composite near metal foil may lead to corrosion. Hence, it is important to preserve the insulating characteristic of epoxy resin for these structures.

Apart from graphene, two-dimensional (2D) transition metal chalcogenides (TMD) [5,6,7,8,9,10] and boron nitride [11,12,13,14,15,16] nanosheets have attracted considerable interest due to their properties similar to graphene. Among these 2D TMD materials, molybdenum disulfide (MoS_2_) has gained special interest due to its applications in the field of electronics [17,18,19], catalysis [20,21,22,23,24], biomedicine [25,26,27], environment [28], and polymer composites [29,30]. Few layered 2D MoS_2_ nanosheets exhibit excellent mechanical properties, such as extremely high Young’s modulus of 0.3 TPa [31] and breaking strength of ~15 N m^−1^ (23 GPa) [32], which are comparable to those of graphene oxide, thus making them potential reinforcing agents for polymer nanocomposites. Another advantage of 2D MoS_2_ nanosheets is their high bandgap that does not impart conductivity to the insulating polymer matrices. These properties make the 2H polytype of MoS_2_ nanosheets an ideal complementary filler to graphene. MoS_2_ has been explored by many researchers for improving the thermal and fire-retardant properties of thermoplastic polymers, such as polypropylene [33,34], polystyrene, and polymethylmethacrylate [29]. To overcome the issue of aggregation nanoplatelets, researchers have tried functionalizing TMDs. There are two main strategies for the functionalization of TMDs, covalent and noncovalent. The covalent modification of graphene oxide (GO) and its derivatives is relatively much easier, unlike TMDs, owing to the presence of various functional groups, such as hydroxyl, epoxy, and carboxylic acid groups on its basal plane. Hence, various methods for covalent modification exist using the coupling reaction between oxygen functionalities and functional groups on the modifier [1,35,36,37,38]. However, for TMDs and specially MoS_2_, covalent functionalization is achieved by the intercalation method. One of the most used methods involves the Li-ion intercalation step followed by treatment with functional molecules [16,39,40]. However, it is to be noted that most of the covalent methods follow two or more steps for functionalization [40].

A few studies have reported using functionalized MoS_2_ for thermosetting polymers such as epoxy [41]. Chhetri et al. used the hydrothermal method for synthesizing CTAB functionalized MoS_2_ platelets leading to the formation of microparticles by self-assembly of nanosheets. The use of these functionalized MoS_2_ demonstrated a 23% increase in the tensile strength [42] of bifunctional epoxy. Zhao et al. showed improvement in corrosion-resistance of epoxy coating on steel by incorporating functionalized MoS_2_ nanosheets [40]. However, this study did not evaluate the bulk properties of the epoxy nanocomposites. Zhao et al. focused on improving the tensile properties of low-temperature bifunctional epoxy using 3-mercaptopropyltriethoxysilane functionalized MoS_2_ nanosheets [41]. The three-step method of intercalation, exfoliation followed by chemical modification using silane functional molecules was employed. The results showed that tensile properties show maximum improvement with the inclusion of 0.7 weight percent of covalently functionalized MoS_2_ nanosheets. Chen et al. covalently modified MoS_2_ using amine-containing melamine for improving the chemical interaction of filler with epoxy for improved tensile properties [43]. Similar to a study by Zhao et al. [41], covalently modified MoS_2_ showed a maximum improvement in properties at 0.8 wt% of filler. The literature available focusing on the effect of MoS_2_ nanosheets on the fracture, compression, and flexural properties of high-performance tetrafunctional epoxy polymers is still inadequate. Eksik et al. [44] reported a 60% improvement in fracture properties of bifunctional epoxy at low loading level (0.2 wt%) of pristine MoS_2_ nanosheets. They concluded that the surface modification of MoS_2_ is required to improve the interaction of the filler with the epoxy matrix. We have reported the effect of surface-functionalized tungsten disulfide (WS_2_) on the fracture, compression, flexural properties, and thermal behavior of epoxy in our previous study. This study explores MoS_2_ as an economically favorable alternative to WS_2_ for enhanced mechanical andthermal properties of epoxy nanocomposites [45].

In this work, we report the effect of surface modified MoS_2_ nanosheets on the fracture, compression, flexural, and thermal behavior of tetrafunctional epoxy polymers. The work involves the one-pot exfoliation and modification of MoS_2_ nanoplatelets at room temperature using cetyltrimethylammonium bromide (CTAB) by bath sonication. The f-MoS_2_ has been incorporated in the range 0.1 wt% to 1 wt% to study and compare the properties of epoxy nanocomposites with neat epoxy polymers.

## 2. Materials and Methods

### 2.1. Materials

Powdered 2H polytype molybdenum disulfide (MoS_2_) was purchased from Sigma Aldrich, Bengaluru, India. Cetyl trimethyl ammonium bromide (CTAB) was purchased from SRL Chemicals, Bengaluru, India. *N*,*N*,*N′*,*N′*-tetraglycidyl-4,4′-methylenebisbenzenamine resin (Epoxy, Araldite MY 721, TGDDM) (Epoxy Value, eq./kg −109 to 116) as well as 4,4′-diaminodiphenylsulfone (DDS, Aradur 9664) powder (curing agent with H^+^ active equivalent of 62 g/eq.) were procured from Huntsman Advanced Materials, The Woodlands, TX, USA. In all the experiments, double autoclaved MilliQ water (Millipore, Billerica, MA, USA) was used.

### 2.2. Preparation of CTAB Exfoliated MoS_2_

In a typical experiment, 900 mg of 2H polytype—MoS_2_ powder was added to 90 mL of 1% aqueous solution of CTAB and subjected to bath sonication for 8 h. The suspension was centrifuged at 15,000 RPM (REMI Instruments, Mumbai, India) for 10 min. The supernatant containing f-MoS_2_ (CTAB functionalized MoS_2_) was separated, further washed (15,000 RPM in a centrifuge) with ethanol and water several times, and dried for a duration of 24 h at 50 °C.

### 2.3. Preparation Epoxy/f-MoS_2_ Nanocomposites

Dried f-MoS_2_ nanoplatelets were dispersed in acetone (10 mg/mL) using a bath sonicator for 2 h. Epoxy was heated to 80 °C, and the required amount of f-MoS_2_ nanoplatelets suspension was added and stirred for 2 h using an overhead stirrer (IKA, Staufen, Germany) followed by mixing using a homogenizer for 10 min. Post mixing, the modified epoxy resin was degassed in a vacuum oven for 12 h at 90 °C to remove the residual solvent molecules. A stoichiometric amount of hardener was added to the mixture at 120 °C assisted with stirring at high speed for 20 min to ensure complete mixing of the epoxy and the hardener. The mixture was again degassed for 2 h at 90 °C to remove the bubbles introduced during the mixing process. Finally, the mixture was cast in a mold (preheated to avoid gas entrapment) and cured at 150 °C for 2 h followed by 180 °C for 2 h followed by 200 °C for 2 h. Nanocomposites containing 0.1, 0.25, 0.5, and 1 wt% of f-MoS_2_ nanoplatelets were fabricated, and for comparison, neat epoxy composites were also fabricated using similar processing conditions.

### 2.4. Characterization

Surface morphology of bulk exfoliated MoS_2_ and fractured epoxy nanocomposites were analyzed by scanning electron microscopy (FEI Sirion XL30, Eindhoven, The Netherlands) Bulk MoS_2_ and f-MoS_2_ nanoplatelets suspensions were prepared in an aqueous medium. Suspensions were diluted and drop cast on a freshly cleaned silicon substrate. Each sample was sputtered to build a 10 nm thick Au layer to prevent charge accumulation during analysis.

The structure and morphology of f-MoS_2_ nanoplatelets were analyzed by transmission electron microscopy (JEOL 2000 FX-II, Akishima, Tokyo, Japan). Nanoplatelets were sonicated in an aqueous medium for 30 min, drop-cast on a Cu grid (carbon-coated), dried and analyzed.

Atomic force microscopy was performed in tapping mode using a NanoWizard 3 AFM (JPK Instruments, Berlin, Germany) to determine the thickness of f-MoS_2_ nanoplatelets. Samples were prepared by sonicating f-MoS_2_ nanoplatelets in deionized water for 30 min, followed by drop-casting on a freshly cleaned silica wafer. Samples were dried and desiccated before conducting AFM analysis.

The photoluminescence properties of the bulk and exfoliated f-MoS_2_ nanoplatelets were evaluated by confocal microscopy (Zeiss LSM 710, Oberkochen, Germany). The powdered samples of the bulk and exfoliated f-MoS_2_ nanoplatelets were mounted on a glass slide and imaged, covering the wavelength from 400 to 700 nm.

FTIR analysis of pure CTAB, bulk MoS_2_ and f-MoS_2_ nanoplatelets were performed on Thermo-Nicolet 6700 Fourier transform infrared spectrophotometer (Thermo Fisher Scientific, Waltham, MA, USA) in the 400–4000 cm^−1^ spectral range. Thermogravimetric analysis was performed on NETZSCH-STA-409 (NETZSCH-Gerätebau GmbH, Selb, Germany) in the range of 30 °C to 800 °C with a heating rate of 10 °C per minute. X-ray powder diffraction was performed for 2θ angles from 8° to 60° using X’Pert PRO (PANalytical, Almelo, The Netherlands).

Dynamic mechanical (DMA) study of nanocomposites was performed on Gabo Eplexor 500N (NETZSCH-Gerätebau GmbH, Selb, Germany) using samples of three-point bend geometry with measurements 5 × 2 × 45 mm^3^. The temperature was gradually changed from 30 to 330 °C at 3 °C min^−1^ with a frequency of 1 Hz.

Compression tests were performed using a Zwick/Rowell Z100 (ZwickRoell, Ulm, Germany) per ASTM D695. Five parallels of each sample with a cylindrical geometry (diameter 6.9 mm, length 10.4 mm) were tested, and the average value is reported here. The cross-sectional area of samples was polished using P4000 grade emery paper to ensure no friction exists between the sample surface and the compression clamps.

The fracture toughness of epoxy nanocomposites was calculated by conducting single edge notch bend tests per ASTM D5045, using a Zwick/Rowell Z100 machine. Samples with dimensions 6 × 12 × 48 mm^3^ were cast molded. A sharp natural crack was induced in the samples by applying sawing motion using a razor blade dipped in liquid N_2_ over the machined notch. The fracture toughness K_IC_ was calculated using the below-mentioned formula:

(1) $$K_{IC}=\frac{P_{\max}}{{\mathrm{BW}}^{1/2}}f(a/W),$$

where P_max_ represents the max load on the load-displacement graph, W is the width of the flexural sample, B is the depth of the sample, and a is the length of pre-crack. The geometry dependent function, f (a/W), is calculated using the following equation:

(2) $$f\left(\frac{a}{W}\right)={6x}^{1/2}\frac{[1.99-x\left(1-x\right)\left(2.15-3.93x+2.7x^{2}\right)]}{\left(1+2x\right){\left(1-x\right)}^{3/2}},$$

Tests were conducted on six replicas per composition, and the average of the obtained values is reported here. The samples were loaded until a complete fracture occurred.

The flexural strength of neat epoxy and modified nanocomposites were determined using a Zwick/Rowell Z100 according to ASTM D790. Five replicas per composition were tested at a strain rate of 2 mm min^−1^, and the average of the values is reported here. Samples with span length 48 mm, thickness 3 mm, and width 12 mm were employed.

## 3. Results and Discussion

### 3.1. Noncovalent Functionalization of MoS_2_ and Characterization of f-MoS_2_ Nanoplatelets

**Figure 1:**
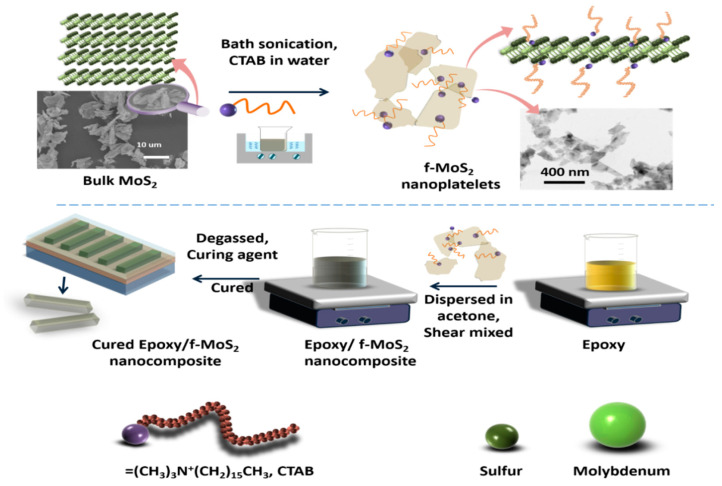
The route to obtaining f- MoS_2_ nanoplatelets and dispersion of f-MoS_2_ nanoplatelets in the epoxy matrix.

MoS_2_ flakes were exfoliated by sonication in an aqueous solution containing CTAB. The long hydrocarbon chains of the surfactant help in exfoliating the stacked layers of MoS_2_ when subjected to bath sonication. The presence of the surfactant molecules also provides the appropriate surface tension to overcome the van der Waals forces between the exfoliated layers. A schematic of the process for the simultaneous noncovalent modification of MoS_2_ with CTAB and exfoliation of f-MoS_2_ nanoplatelets followed by the fabrication of f-MoS_2_ modified epoxy nanocomposites is shown in Figure 1. The CTAB modified MoS_2_ nanoplatelets disperse quickly in a weakly polar solvent such as acetone due to the polar nature of the CTAB molecules.

**Figure 2:**
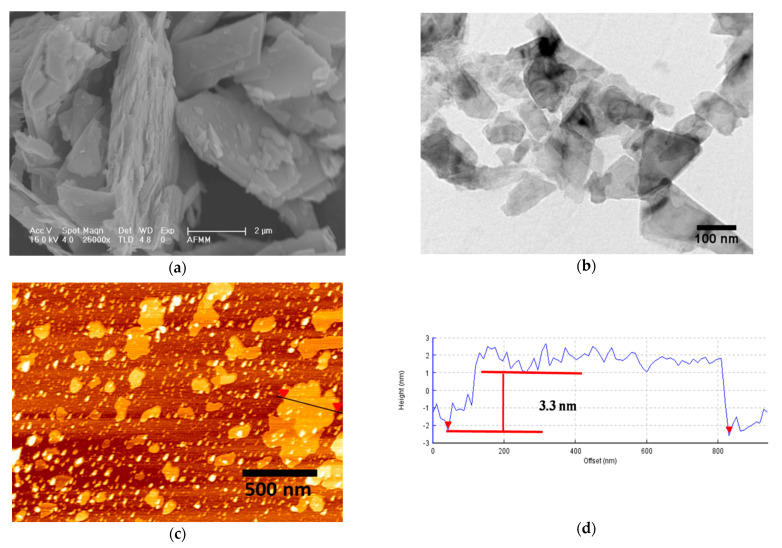
(**a**) SEM micrograph of bulk MoS_2_ platelets, (**b**) TEM image of exfoliated f-MoS_2_ nanoplatelets. (**c**) AFM image of the exfoliated f-MoS_2_ nanoplatelets. (**d**) Height profile of the exfoliated f-MoS_2_ nanoplatelets.

Figure 2a shows the SEM micrographs of bulk MoS_2_. The particles were a few microns in size. The exfoliated layers of f-MoS_2_ are visible in the TEM micrographs (Figure 2b), and the sheets now appear to be about 100 nm. The AFM image and height profile of f-MoS_2_ are shown in Figure 2c,d. The height profile revealed the thickness of nanoplatelets to be 3.3 nm (Figure 2d). A monolayer of MoS_2_ is approximately 0.69 nm thick [32]. The thickness of nanoplatelets obtained is almost five times the thickness of the monolayer. Therefore, exfoliation using CTAB, and sonication offers a simple approach to produce thinner sheets of MoS_2_.

**Figure 3:**
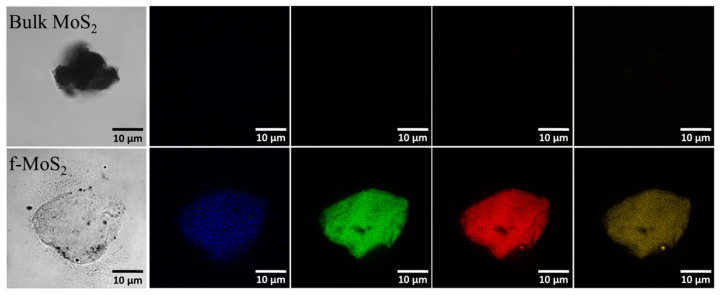
Confocal images of bulk MoS_2_ and exfoliated f-MoS_2_ nanoplatelets excited from 400–700 nm range (Blue to Far-red).

Confocal microscopy images of the bulk and exfoliated MoS_2_ flakes (Figure 3) demonstrated that exfoliated MoS_2_ exhibits excitation-dependent emission photoluminescence, which is not observed in the bulk MoS_2_. The photoluminescence properties of the exfoliated f-MoS_2_ nanoplatelets are mainly due to the intrinsic electronic properties of the monolayer material and Eda et al. [46] have reported similar results. The absence of photoluminescence in the bulk material is mainly due to the indirect bandgap of the electronic transition and its higher refractive index that causes a decrease in the local electric field. The transition of bulk MoS_2_ to exfoliated MoS_2_ nanoplatelets shifts the photoluminescence properties due to a change in the direct gap electronic transition of MoS_2_. It also increases the local electric field due to a decrease in the refractive index [47].The excitation-dependent emission photoluminescence property from the blue region to the far-red region can be attributed to the polydispersity of the 2D layers and quantum size effect, which is not observed in the bulk material [48,49].

**Figure 4:**
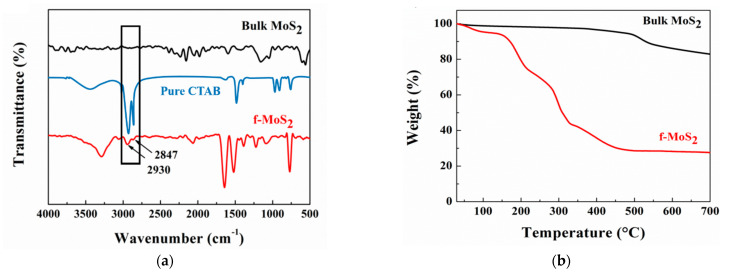
(**a**) FTIR spectra pure CTAB, bulk MoS_2_ and exfoliated f-MoS_2_ nanoplatelets (**b**) TGA curves of bulk MoS_2_ and exfoliated f-MoS_2_ nanoplatelets.

FTIR spectroscopy was used to confirm the successful noncovalent organic modification of MoS_2_ nanoplatelets layers with CTAB (Figure 4a). In addition to the peaks observed for bulk MoS_2_, the absorption peaks at 2847 cm^−1^ and 2930 cm^−1^ in the spectrum of f-MoS_2_ can be ascribed to CH_3_ and CH_2_ groups of CTAB molecules, respectively. The strong peaks at 1642 cm^−1^ and 1445 cm^−1^ can be attributed to the presence of CH_3_-N^+^ symmetric vibration of CTAB.

Thermogravimetric analysis was performed to further confirm the noncovalent functionalization of CTAB molecules on MoS_2_ nanoplatelets. The thermal decomposition curves are shown in Figure 4b. Bulk MoS_2_ lost 9% of the total weight, similar to the weight percent of SO_2_ (10 wt%) [50]. Hence the observation suggests that in air, bulk MoS_2_ transforms to molybdenum oxide. In the case of f-MoS_2_, the observed weight loss was much higher in the temperature range of 100–700 °C when compared to bulk MoS_2_. For example, the temperature for 5 percent weight loss (T_−5%_) was 114 °C for f-MoS_2_, whereas T_−5%_ was much higher at 497 °C for bulk MoS_2_ due to the absence of organic moieties. The residual mass for bulk MoS_2_ was 95 wt%, whereas f-MoS_2_ showed 27 wt% residual mass at 700 °C. The increased weight loss can be ascribed to the increased diffusion of heat in exfoliated layers and decomposition of CTAB moieties, thus confirming the successful functionalization of MoS_2_ nanoplatelets.

**Figure 5:**
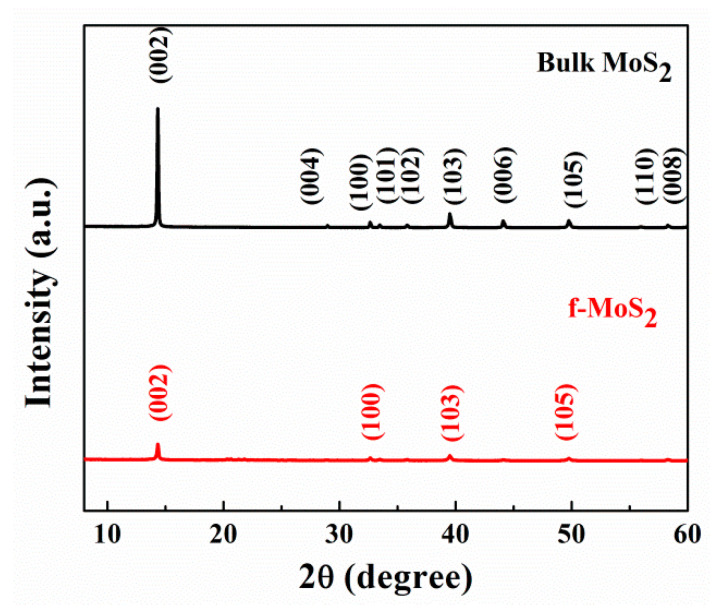
XRD spectra of bulk MoS_2_ and exfoliated f-MoS_2_ nanoplatelets.

The effect of exfoliation and surface functionalization on the crystalline structure of MoS_2_ was studied by X-ray diffraction (XRD) analysis. Figure 5 shows the XRD graph of the bulk MoS_2_ and f-MoS_2_ nanoplatelets. The sharp and intense characteristic peak of bulk MoS_2_ appeared at 14.35°, which indicated interplanar spacing of 0.62 nm and was attributed to the (002) plane of the bulk MoS_2_. Other peaks appeared at 28.98, 32.63, 33.46, 39.50, 44.08, and 49.75°, which corresponded to the (004), (100), (101), (103), (006), and (105) planes, respectively. All the peaks were in good agreement with the JCPDS data for the bulk MoS_2_ (card no. 24-0513). In the case of f-MoS_2_, the diffraction peaks for the planes (104), (106), and (108) disappeared. The intensity of peak (002) of exfoliated MoS_2_ reduced dramatically compared to the bulk MoS_2_. This might be attributed to the reduced crystallite size, which reduces the probability of several (002) planes to satisfy Bragg’s condition. Additionally, peaks representing (004), (100), (101), (103), (006), and (105) also showed a drastic reduction in intensity compared to bulk MoS_2_. The reduced intensity of the peak (002) indicates the successful exfoliation of MoS_2_.

### 3.2. Thermal Properties of Epoxy/f-MoS_2_ Nanocomposites

#### 3.2.1. Thermogravimetric Analysis

**Figure 6:**
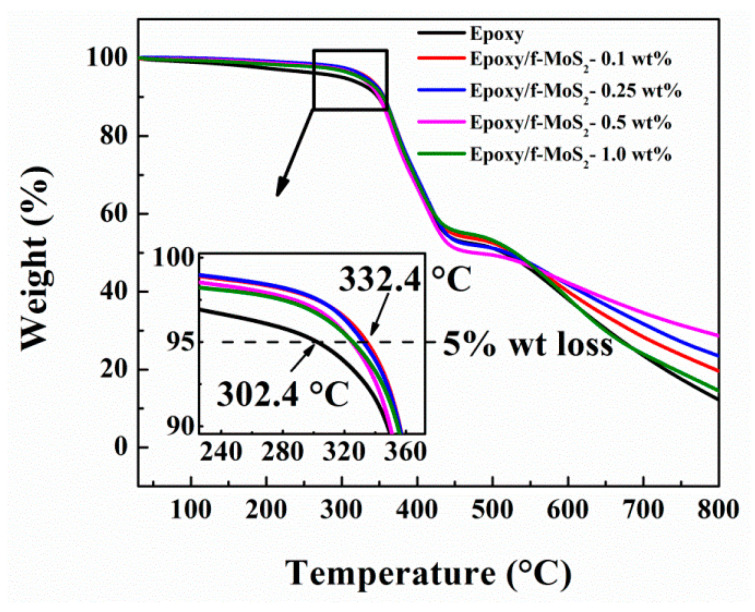
TGA curves of the Epoxy and Epoxy/f-MoS_2_ nanocomposites.

The inorganic layered nanofillers are expected to affect the thermal performance of nanocomposites to a significant extent due to their excellent thermal resistance. The thermal performance of nanocomposites was measured by thermogravimetric analysis. Figure 6 shows the curves from the thermogravimetric analysis of neat epoxy and Epoxy/f-MoS_2_ nanocomposites, and the crucial data are shown in Table 1. For all the fabricated nanocomposites, thermal decomposition happens in one step. The most significant weight loss (~60%) occurred in the temperature range of 300–450 °C due to the gradual degradation of the epoxy crosslinked network. The addition of f-MoS_2_ nanoplatelets increased the T_−5%_ of the epoxy nanocomposites by 20–30 °C. For example, the nanocomposite with 0.25 wt.% of f-MoS_2_ shows T_−5%_ at 332°C, which is greater than T_−5%_ of neat epoxy at 302 °C. This improvement in thermal properties can be ascribed to the barrier effect of well dispersed and exfoliated f-MoS_2_ nanoplatelets, which restrict the mobility of the volatile products across the matrix. The presence of CTAB molecules enhances the affinity of the MoS_2_ nanoplatelets towards the epoxy resulting in better dispersion of the f-MoS_2_ nanoplatelets in the epoxy matrix. The large surface area of MoS_2_ nanoplatelets, along with CTAB assisted dispersion, paves the way for the improved interlocking of filler with epoxy matrix.

Hence, the f-MoS_2_ nanoplatelets delay the degradation process by restricting the diffusion of degradation products and heat due to enhanced interface. The char yield is an essential factor for assessing the thermal stability of the polymer nanocomposite as the char residue resists heat flow during combustion and helps improve thermal stability. Hence, the higher the char yield, the better the thermal stability of nanocomposites [33,51]. The char yields of all the modified nanocomposites also shows significant improvement. Hence, it can be concluded that f-MoS_2_ nanoplatelets enhance the thermal stability of epoxy nanocomposites. For example, the nanocomposite with 0.5 wt% filler yielded 29% char, which is much larger than the char yield of 12% for the neat epoxy. It can be noted that epoxy nanocomposites with 1.0 wt% show the least improvement in thermal stability that can be ascribed to the poor dispersion of f-MoS_2_ nanosheets at high weight % loading. The presence of agglomerates also hinders the nanofiller from forming a stronger interface and imparting improved thermal properties.

#### 3.2.2. Dynamic Mechanical Analysis

**Figure 7:**
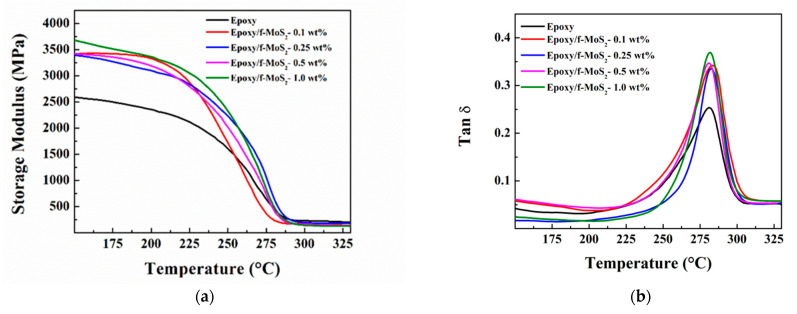
Storage modulus (**a**) and tan δ (**b**) vs. temperature of Epoxy & Epoxy/f-MoS_2_ nanocomposites.

The dynamic mechanical study was employed to study the effect of f-MoS_2_ nanoplatelets on the thermomechanical properties of nanocomposites. The storage modulus and tan δ curves of the nanocomposites and the neat epoxy are shown in Figure 7a,b. Since tetrafunctional epoxy polymers are the choice for high-temperature applications due to their high (>200 °C) glass transition temperature (T_g_), the epoxy nanocomposites must retain enhanced mechanical properties at elevated temperatures. Figure 7a shows the storage modulus of epoxy nanocomposites at various temperatures, including temperatures below and above T_g_ of the nanocomposites. The storage modulus is a measure of the stiffness of the nanocomposites. The storage modulus of the nanocomposite Epoxy/f-MoS_2_-0.25 wt% showed a maximum increase of 28% at 200 °C and 39% at 250 °C in comparison to neat epoxy. T_g_ of the polymer nanocomposites is taken as the temperature at the maximum of the tan δ curve. Figure 7b shows the change in tan δ with temperature for the neat epoxy and epoxy modified with different loading of f-MoS_2_ nanoplatelets. The epoxy nanocomposites modified with low levels (0.1 and 0.25 wt%) of f-MoS_2_ nanoplatelets showed a higher T_g_ than the neat epoxy (281.1 °C) with a higher value of storage modulus. The increase in T_g_ suggests improvement in the thermo-mechanical properties of the final nanocomposites. The increase in storage modulus values and T_g_ for epoxy with the loading of f-MoS_2_ nanoplatelets should be attributed to the robust nature and high intrinsic stiffness of the MoS_2_ nanoplatelets. The segmental motional is restricted by the large aspect ratio of f-MoS_2_ platelets. The presence of CTAB molecules over the surface of the MoS_2_ nanoplatelets provides enhanced compatibility with the epoxy matrix due to its long hydrocarbon chains. Better exfoliation and distribution of the f-MoS_2_ in the epoxy matrix lead to enhanced effective volume of the filler, which is also one of the factors for the excellent reinforcing effect of f-MoS_2_ nanoplatelets.

### 3.3. Mechanical Properties of Epoxy/f-MoS_2_ Nanocomposites

#### 3.3.1. Compressive Properties

**Figure 8:**
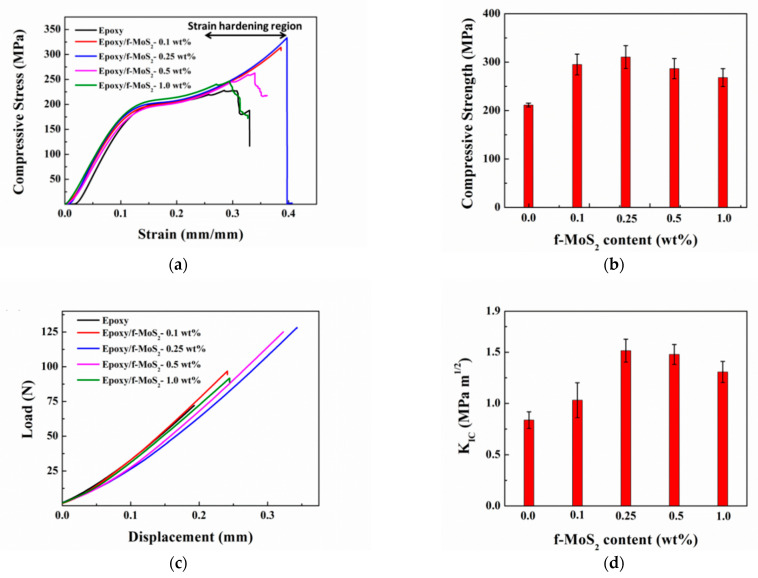
(**a**) Typical true stress versus strain curves for compression test and (**b**) compressive strength versus f-MoS_2_ content of the Epoxy and Epoxy/f-MoS_2_ nanocomposites. (**c**) Typical load-displacement curves from fracture toughness test & (**d**) fracture toughness vs. f-MoS_2_ content of the Epoxy and Epoxy/f-MoS_2_ nanocomposites.

Figure 8a shows the typical true stress vs. strain curves for compression tests for the neat epoxy and epoxy modified with f-MoS_2_ nanoplatelets with loading up to 1 wt%. All the samples showed similar deformation behavior under the applied load with an initial elastic region up to the diffused yield zone followed by the strain hardening region until the sample fractures. The strain hardening region is an indication of the energy absorbed by the sample before fracture. Unlike in instances of rubber toughened epoxy, a strain-softening region was not observed for any of the epoxy samples. Strain softening causes enhancement in the local cross-sectional area, which stabilizes further local strain [52]. The absence of strain-softening region is attributed to the high crosslink density of tetrafunctional epoxy & intrinsic strength of f-MoS_2_ nanoplatelets, which leads to stronger composites. It can be noted in Figure 8a that the strain hardening region increased for all the epoxy samples modified with f-MoS_2_ nanoplatelets. However, the extent of variation was different for different loading of filler. Nanocomposites with 0.25 wt% of f-MoS_2_ nanoplatelets showed the most significant increase in the strain hardening region and are, therefore, most effective in absorbing the stress during the compressive loading. Figure 8b shows the comparative plot of compressive strengths of neat epoxy and epoxy nanocomposites. The compressive strength increased as the content of f-MoS_2_ increased from 0.1 wt% to 0.25 wt%. The addition of 0.25 wt% of f-MoS_2_ nanoplatelets showed the maximum improvement in compressive strength compared to epoxy nanocomposites loaded with higher wt% of f-MoS_2_ nanoplatelets (0.5 to 1.0 wt%). For example, the incorporation of 0.25 wt% of f-MoS_2_ nanoplatelets in epoxy leads to a 46% improvement in compressive strength of the nanocomposite in comparison to the neat one. The enhancement in the compressive properties could be ascribed to the high intrinsic strength and good dispersion of f-MoS_2_ nanoplatelets caused by organic CTAB moieties, which promote enhanced interaction with the epoxy network and reduce agglomeration. The effective concentration of f-MoS_2_ improves due to uniform dispersion of the filler owing to organic modification of its surface. Uniform dispersion gives rise to better load transfer from epoxy matrix to f-MoS_2_ nanoplatelets during the compressive loading. Nanocomposites with 1 wt% of f-MoS_2_ nanoplatelets show a reduced level of improvement with 26% enhancement in the compressive properties. This reduction could be ascribed to the reduced degree of distribution of f-MoS_2_ nanoplatelets at higher loading levels.

#### 3.3.2. Fracture Properties

The fracture properties of neat epoxy and Epoxy/f-MoS_2_ nanocomposites loaded with 0.1, 0.25, 0.5, and 1 wt% of filler, obtained by performing a three-point bend test on notched samples, are shown in Figure 8c,d. Typical curves of load versus displacement are shown in Figure 8c. Figure 8d shows the base value of fracture toughness of neat epoxy to be 0.84 MPa m^1/2^. It is evident that the presence of f-MoS_2_ nanoplatelets significantly improves the fracture properties of the nanocomposites. The maximum improvement in the fracture properties was observed with an f-MoS_2_ nanoplatelets loading of 0.25 wt% as it shows K_IC_ value of 1.52 MPa m^1/2^. This value corresponds to an improvement of 81% when compared to neat epoxy. The extremely high Young’s modulus of MoS_2_ nanoplatelets [31], which is higher than that of graphene oxide, is one of the primary reasons for the improved fracture properties of the f-MoS_2_ modified epoxy nanocomposites. MoS_2_ nanosheets are intrinsically stiff and provide excellent barrier properties for crack propagation. The toughening effect is further improved by the enhanced compatibility of organically modified MoS_2_ nanoplatelets with the epoxy matrix. The long chains of CTAB cause hindrance between the layers due to its bulky nature, promoting exfoliation and avoiding the agglomeration of layers. CTAB modification also makes MoS_2_ layers more compatible with the epoxy matrix. This enhanced affinity of the MoS_2_ nanoplatelets leads to an even stronger interface which helps in better stress transfer from the epoxy matrix to f-MoS_2_ nanoplatelets during mechanical loading. The large surface area of MoS_2_ nanoplatelets enables interlocking of the MoS_2_ nanoplatelets with epoxy over a much larger interfacial area. The modification of the nanoplatelets with the organic molecules of CTAB helps realize the full potential of the MoS_2_ nanoplatelets as a toughening agent for epoxy. As the content of f-MoS_2_ nanoplatelets reaches 1 wt%, the degree of improvement dips to 56%. Thus, f-MoS_2_ nanoplatelets provide maximum enhancement in fracture properties at lower loading levels of 0.25 wt%. The decrease in toughening effect with loading beyond 0.25 wt% could be ascribed to the increased agglomeration of nanoplatelets in the epoxy at a higher concentration level. Agglomeration leads to a reduction in the interfacial area, thus limiting the interaction of the f-MoS_2_ nanoplatelets with the epoxy matrix. The presence of agglomerates also increases defects, which reduces the intrinsic cured properties of the epoxy composite. Further analysis of fractured samples obtained from the fracture toughness test was done by scanning electron microscopy.

#### 3.3.3. Study of Fractured Surfaces

**Figure 9:**
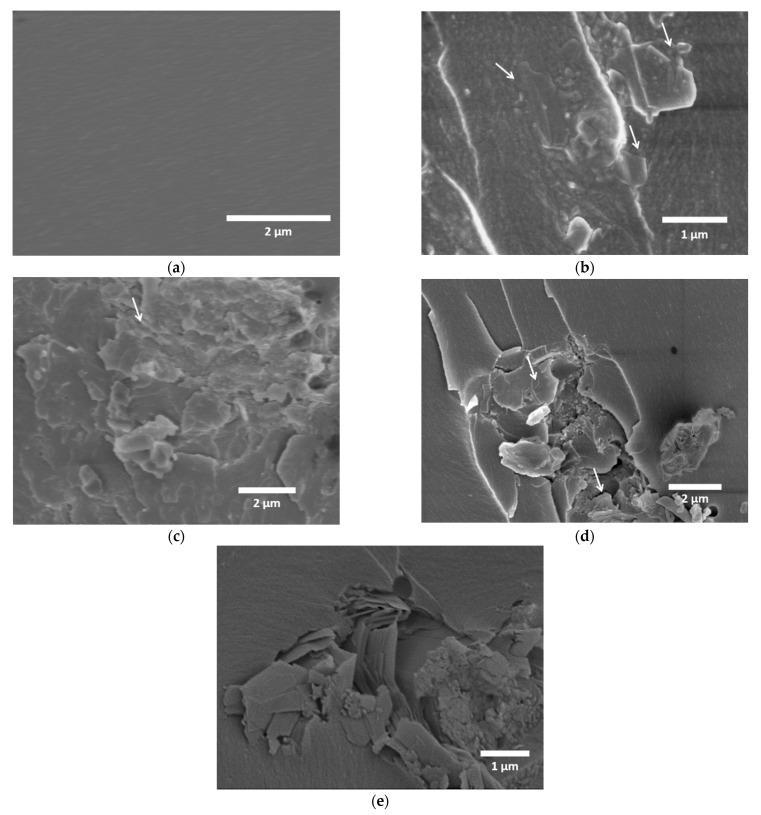
SEM images of fractured sections of (**a**) Epoxy, (**b**) Epoxy/f-MoS_2_-0.1 wt%, (**c**) Epoxy/f-MoS_2_-0.25 wt%, (**d**) Epoxy/f-MoS_2_-0.5 wt% and (**e**) Epoxy/f-MoS_2_-1.0 wt%.

The homogeneous dispersion of the nano-reinforcements in the polymer composites provides a maximum enhancement in the mechanical properties of polymer composites. The dispersion state of f-MoS_2_ nanoplatelets in the epoxy matrix was analyzed using SEM. Figure 9 shows the SEM micrographs of the cross-sectional areas of the fractured surfaces of neat epoxy and Epoxy/f-MoS_2_ nanocomposites of different compositions. The neat epoxy had a smoother and featureless surface (Figure 9a), while the modified epoxy nanocomposite with f-MoS_2_ layers showed an irregular surface (Figure 9b–e). It can be seen in Figure 9b–d that f-MoS_2_ nanoplatelets cause less agglomeration in the epoxy nanocomposites owing to the lower filler content. The improved dispersion of f-MoS_2_ nanoplatelets leads to a stronger interface between filler and the matrix, thus enhancing the nanocomposites’ mechanical properties. The enhanced interfacial characteristics of epoxy modified with 0.25 wt% of f-MoS_2_ may also be attributed to the thin structure of exfoliated nanoplatelets resulting in wrinkled morphology, thus enabling better interlocking between the reinforcing agent and the epoxy polymer chains. The micrographs show that the filler has excellent adhesion to the matrix as no pullout or loose nanoplatelets were observed. The enhanced compatibility of the f-MoS_2_ nanoplatelets could be ascribed to the presence of organic CTAB molecules on the surface of the MoS_2_ nanoplatelets. However, as the f-MoS_2_ content reached 1 wt%, it is observed that the distribution of the filler in the matrix becomes non-uniform (Figure 9e). The agglomeration and non-homogeneous distribution of the nanoplatelets in the epoxy matrix reduce the interfacial area and the degree of interaction between f-MoS_2_ nanoplatelets and epoxy, reducing the efficiency of the filler as a reinforcing agent. The agglomerated MoS_2_ also act as a defect hindering the crosslinking of epoxy and hardener. The agglomeration does not significantly improve the mechanical properties of epoxy nanocomposites with 1 wt% of the filler.

#### 3.3.4. Flexural Properties

**Figure 10:**
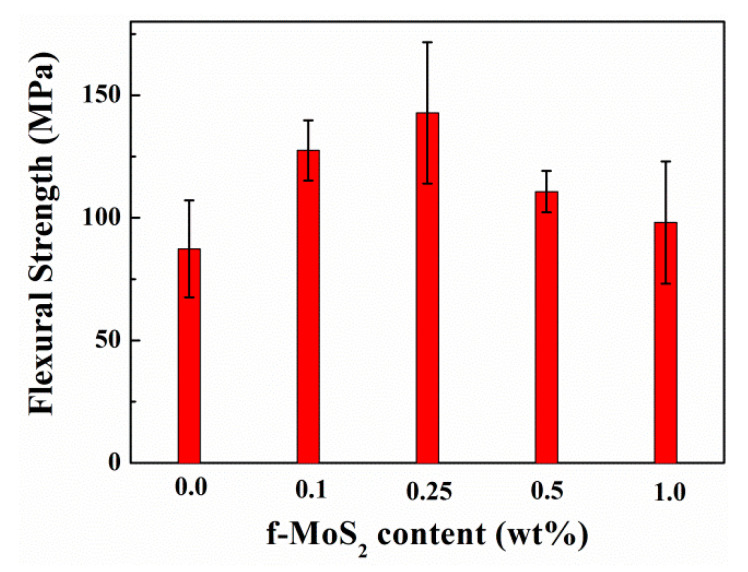
Flexural strength vs. f-MoS_2_ content of the Epoxy and Epoxy/f-MoS_2_ nanocomposites.

The flexural properties of neat epoxy and Epoxy/f-MoS_2_ nanocomposites are compared in Figure 10. F-MoS_2_ nanoplatelets improved the flexural properties of epoxy nanocomposites for the entire range of loading percentage, although the degree of improvement varied with the loading level. Like the trend observed in the compressive and the fracture properties, nanocomposites modified with 0.25 wt% of f-MoS_2_ nanoplatelets give rise to a maximum increase of 64% in flexural strength compared to the neat epoxy. The flexural strength of the Epoxy/f-MoS_2_-0.25 wt% corresponds to 143 MPa, which is significantly high compared to the flexural strength of 87 MPa observed for the neat epoxy. The results are consistent with the fracture and compressive properties of the nanocomposites. The improvement in flexural properties is minimal beyond 0.25 wt% of platelets. The least improvement is observed with 1 wt% of f-MoS_2_ nanoplatelets, which can be attributed to the poor dispersion of the nanoplatelets in the matrix. The results confirm that f-MoS_2_ nanoplatelets show better improvement in the flexural properties at low loading levels because of the better dispersion and stronger interface facilitated by the presence of CTAB molecules.

## 4. Conclusions

In conclusion, 2D MoS_2_ nanoplatelets were successfully modified using a noncovalent method for the enhanced fracture and thermal properties of tetrafunctional epoxy nanocomposites. Organic modification supports better compatibility, dispersion, and interaction of the MoS_2_ nanoplatelets with the epoxy matrix. f-MoS_2_ nanoplatelets were prepared by the one-pot sonication method. The inclusion of f-MoS_2_ nanoplatelets in relatively low amounts induced an excellent improvement in fracture, flexural, compression, and thermal properties. The remarkable improvement in the mechanical properties was attributed to better interfacial adhesion between f-MoS_2_ nanoplatelets and the epoxy matrix and a better distribution of the layers due to the presence of the bulky organic groups on the nanoplatelets surface. The loading of f-MoS_2_ nanoplatelets beyond 0.25 wt% leads to a relatively non-homogeneous distribution of the nanoplatelets in the epoxy matrix, causing a reduction in the extent of interaction and the interfacial area between the filler and the matrix. This work demonstrates the potential of organically modified MoS_2_ nanosheets as a toughening agent for tetrafunctional epoxy nanocomposites.

## Figures and Tables

**Table 1:** Thermomechanical properties of the Epoxy and Epoxy/f-MoS_2_ nanocomposites.

Sample	T_g_^a^ (°C)	T_−5%_^b^ (°C)	Char Yield (%) ^c^	Storage ^d^ Modulus @ 200 °C (MPa)	Storage ^d^ Modulus @ 250 °C (MPa)
Epoxy	281.1	302	12.2	2357	1606
Epoxy/f-MoS_2_-0.1wt%	283.5	335	19.7	3332	1741
Epoxy/f-MoS_2_-0.25 wt%	282.3	332	23.5	3101	2229
Epoxy/f-MoS_2_-0.5 wt%	280.9	325	29.0	3187	2010
Epoxy/f-MoS_2_-1.0 wt%	281.6	326	14.2	3367	2338

^a^ T_g_—Glass transition temperature. ^b^ T_−5%_—5% weight loss-temperature. ^c^ At 800 °C. ^d^ DMA results of the Epoxy and Epoxy/f-MoS_2_ nanocomposites.

## Data Availability

The datasets generated during and/or analyzed during the current study are available from the corresponding author on reasonable request.

## References

[1] Sahu M., Raichur A.M. (2019). Toughening of high performance tetrafunctional epoxy with poly(allyl amine) grafted graphene oxide. Compos. Part B Eng..

[2] Li Y., Zhang H., Porwal H., Huang Z., Bilotti E., Peijs T. (2017). Mechanical, electrical and thermal properties of in-situ exfoliated graphene/epoxy nanocomposites. Compos. Part A Appl. Sci. Manuf..

[3] Neto A.C., Guinea F., Peres N.M., Novoselov K.S., Geim A.K. (2009). The electronic properties of graphene. Rev. Mod. Phys..

[4] Jia J., Sun X., Lin X., Shen X., Mai Y.-W., Kim J.-K. (2014). Exceptional Electrical Conductivity and Fracture Resistance of 3D Interconnected Graphene Foam/Epoxy Composites. ACS Nano.

[5] Zhou L., He B., Yang Y., He Y. (2014). Facile approach to surface functionalized MoS_2_ nanosheets. RSC Adv..

[6] Backes C., Berner N.C., Chen X., Lafargue P., LaPlace P., Freeley M., Duesberg G.S., Coleman J.N., McDonald A.R. (2015). Functionalization of Liquid-Exfoliated Two-Dimensional 2H-MoS_2_. Angew. Chem. Int. Ed..

[7] Gupta A., Sakthivel T., Seal S. (2015). Recent development in 2D materials beyond graphene. Prog. Mater. Sci..

[8] Han S.A., Bhatia R., Kim S.-W. (2015). Synthesis, properties and potential applications of two-dimensional transition metal dichalcogenides. Nano Converg..

[9] Song I., Park C., Choi H.C. (2015). Synthesis and properties of molybdenum disulphide: From bulk to atomic layers. RSC Adv..

[10] Zhang H. (2015). Ultrathin Two-Dimensional Nanomaterials. ACS Nano.

[11] Zhi C., Bando Y., Tang C., Kuwahara H., Golberg D. (2009). Large-scale fabrication of boron nitride nanosheets and their utilization in polymeric composites with improved thermal and mechanical properties. Adv. Mater..

[12] Golberg D., Bando Y., Huang Y., Terao T., Mitome M., Tang C., Zhi C. (2010). Boron nitride nanotubes and nanosheets. ACS Nano.

[13] Coleman J.N., Lotya M., O’Neill A., Bergin S.D., King P.J., Khan U., Young K., Gaucher A., De S., Smith R.J. (2011). Two-dimensional nanosheets produced by liquid exfoliation of layered materials. Science.

[14] Peng Q., Ji W., De S. (2012). Mechanical properties of the hexagonal boron nitride monolayer: Ab initio study. Comput. Mater. Sci..

[15] Khan U., May P., O’Neill A., Bell A.P., Boussac E., Martin A., Semple J., Coleman J.N. (2013). Polymer reinforcement using liquid-exfoliated boron nitride nanosheets. Nanoscale.

[16] Feng X., Xing W., Liu J., Qiu S., Hu Y., Liew K.M. (2016). Reinforcement of organo-modified molybdenum disulfide nanosheets on the mechanical and thermal properties of polyurethane acrylate films. Compos. Sci. Technol..

[17] Radisavljevic B., Radenovic A., Brivio J., Giacometti V., Kis A. (2011). Single-layer MoS_2_ transistors. Nat. Nanotechnol..

[18] Yin Z., Li H., Li H., Jiang L., Shi Y., Sun Y., Lu G., Zhang Q., Chen X., Zhang H. (2011). Single-layer MoS_2_ phototransistors. ACS Nano.

[19] Lee H.S., Min S.W., Park M.K., Lee Y.T., Jeon P.J., Kim J.H., Ryu S., Im S. (2012). MoS_2_ Nanosheets for Top-Gate Nonvolatile Memory Transistor Channel. Small.

[20] Chianelli R.R., Siadati M.H., De la Rosa M.P., Berhault G., Wilcoxon J.P., Bearden R., Abrams B.L. (2006). Catalytic properties of single layers of transition metal sulfide catalytic materials. Catal. Rev..

[21] Li Y., Wang H., Xie L., Liang Y., Hong G., Dai H. (2011). MoS_2_ nanoparticles grown on graphene: An advanced catalyst for the hydrogen evolution reaction. J. Am. Chem. Soc..

[22] Xiang Q., Yu J., Jaroniec M. (2012). Synergetic effect of MoS_2_ and graphene as cocatalysts for enhanced photocatalytic H_2_ production activity of TiO_2_ nanoparticles. J. Am. Chem. Soc..

[23] Kabachii Y.A., Golub A.S., Kochev S.Y., Lenenko N.D., Abramchuk S.S., Antipin M.Y., Valetsky P.M., Stein B.D., Mahmoud W.E., Al-Ghamdi A.A. (2013). Multifunctional Nanohybrids by Self-Assembly of Monodisperse Iron Oxide Nanoparticles and Nanolamellar MoS_2_ Plates. Chem. Mater..

[24] Kim J., Byun S., Smith A.J., Yu J., Huang J. (2013). Enhanced electrocatalytic properties of transition-metal dichalcogenides sheets by spontaneous gold nanoparticle decoration. J. Phys. Chem. Lett..

[25] Zhu C., Zeng Z., Li H., Li F., Fan C., Zhang H. (2013). Single-layer MoS_2_-based nanoprobes for homogeneous detection of biomolecules. J. Am. Chem. Soc..

[26] Feng W., Chen L., Qin M., Zhou X., Zhang Q., Miao Y., Qiu K., Zhang Y., He C. (2015). Flower-like PEGylated MoS_2_ nanoflakes for near-infrared photothermal cancer therapy. Sci. Rep..

[27] Yin W., Yu J., Lv F., Yan L., Zheng L.R., Gu Z., Zhao Y. (2016). Functionalized nano-MoS_2_ with peroxidase catalytic and near-infrared photothermal activities for safe and synergetic wound antibacterial applications. ACS Nano.

[28] Li J., Liu X., Pan L., Qin W., Chen T., Sun Z. (2014). MoS_2_–reduced graphene oxide composites synthesized via a microwave-assisted method for visible-light photocatalytic degradation of methylene blue. RSC Adv..

[29] Zhou K., Liu J., Zeng W., Hu Y., Gui Z. (2015). In situ synthesis, morphology, and fundamental properties of polymer/MoS_2_ nanocomposites. Compos. Sci. Technol..

[30] Feng X., Wen P., Cheng Y., Liu L., Tai Q., Hu Y., Liew K.M. (2016). Defect-free MoS_2_ nanosheets: Advanced nanofillers for polymer nanocomposites. Compos. Part A Appl. Sci. Manuf..

[31] Castellanos-Gomez A., Poot M., Steele G.A., van der Zant H.S., Agraït N., Rubio-Bollinger G. (2012). Elastic properties of freely suspended MoS_2_ nanosheets. Adv. Mater..

[32] Bertolazzi S., Brivio J., Kis A. (2011). Stretching and breaking of ultrathin MoS_2_. ACS Nano.

[33] Wang D., Song L., Zhou K., Yu X., Hu Y., Wang J. (2015). Anomalous nano-barrier effects of ultrathin molybdenum disulfide nanosheets for improving the flame retardance of polymer nanocomposites. J. Mater. Chem. A.

[34] Feng X., Wang B., Wang X., Wen P., Cai W., Hu Y., Liew K.M. (2016). Molybdenum disulfide nanosheets as barrier enhancing nanofillers in thermal decomposition of polypropylene composites. Chem. Eng. J..

[35] Amirbeygi H., Khosravi H., Tohidlou E. (2019). Reinforcing effects of aminosilane-functionalized graphene on the tribological and mechanical behaviors of epoxy nanocomposites. J. Appl. Polym. Sci..

[36] Li W., Song B., Zhang S., Zhang F., Liu C., Zhang N., Yao H., Shi Y. (2020). Using 3-Isocyanatopropyltrimethoxysilane to decorate graphene oxide with nano-titanium dioxide for enhancing the anti-corrosion properties of epoxy coating. Polymers.

[37] Fang M., Zhang Z., Li J., Zhang H., Lu H., Yang Y. (2010). Constructing hierarchically structured interphases for strong and tough epoxy nanocomposites by amine-rich graphene surfaces. J. Mater. Chem..

[38] Li Z., Wang R., Young R.J., Deng L., Yang F., Hao L., Jiao W., Liu W. (2013). Control of the functionality of graphene oxide for its application in epoxy nanocomposites. Polymer.

[39] Presolski S., Pumera M. (2016). Covalent functionalization of MoS_2_. Mater. Today.

[40] Zhao X., Zhang B., Jin Z., Chen C., Zhu Q., Hou B. (2016). Epoxy coating modified by 2D MoS_2_/SDBS: Fabrication, anticorrosion behaviour and inhibition mechanism. Rsc Adv..

[41] Zhao M., Liu L., Zhang B., Sun M., Zhang X., Zhang X., Li J., Wang L. (2018). Epoxy composites with functionalized molybdenum disulfide nanoplatelet additives. RSC Adv..

[42] Chhetri S., Adak N.C., Samanta P., Mandal N., Kuila T., Murmu N.C. (2018). Investigation of mechanical and thermal properties of the cetyltrimethylammonium bromide functionalized molybdenum disulfide (MoS_2_)/epoxy composites. Polym. Bull..

[43] Chen B., Ni B.-J., Liu W.-T., Ye Q.-Y., Liu S.-Y., Zhang H.-X., Yoon K.-B. (2018). Mechanical properties of epoxy nanocomposites filled with melamine functionalized molybdenum disulfide. RSC Adv..

[44] Eksik O., Gao J., Shojaee A.S., Thomas A., Chow P., Bartolucci S.F., Lucca D.A., Koratkar N. (2014). Epoxy Nanocomposites with Two-Dimensional Transition Metal Dichalcogenide Additives. ACS Nano.

[45] Sahu M., Narashimhan L., Prakash O., Raichur A.M. (2017). Noncovalently Functionalized Tungsten Disulfide Nanosheets for Enhanced Mechanical and Thermal Properties of Epoxy Nanocomposites. ACS Appl. Mater. Interfaces.

[46] Eda G., Yamaguchi H., Voiry D., Fujita T., Chen M., Chhowalla M. (2011). Photoluminescence from chemically exfoliated MoS_2_. Nano Lett..

[47] Splendiani A., Sun L., Zhang Y., Li T., Kim J., Chim C.-Y., Galli G., Wang F. (2010). Emerging photoluminescence in monolayer MoS_2_. Nano Lett..

[48] Ou J.Z., Chrimes A.F., Wang Y., Tang S.-Y., Strano M.S., Kalantar-zadeh K. (2014). Ion-driven photoluminescence modulation of quasi-two-dimensional MoS_2_ nanoflakes for applications in biological systems. Nano Lett..

[49] Wang N., Wei F., Qi Y., Li H., Lu X., Zhao G., Xu Q. (2014). Synthesis of strongly fluorescent molybdenum disulfide nanosheets for cell-targeted labeling. ACS Appl. Mater. Interfaces.

[50] Zhou K., Zhang Q., Liu J., Wang B., Jiang S., Shi Y., Hu Y., Gui Z. (2014). Synergetic effect of ferrocene and MoS_2_ in polystyrene composites with enhanced thermal stability, flame retardant and smoke suppression properties. RSC Adv..

[51] Zhou K., Liu J., Shi Y., Jiang S., Wang D., Hu Y., Gui Z. (2015). MoS_2_ nanolayers grown on carbon nanotubes: An advanced reinforcement for epoxy composites. ACS Appl. Mater. Interfaces.

[52] Haward R.N. (2012). The Physics of Glassy Polymers.

